# Three dimensional printed macroporous polylactic acid/hydroxyapatite composite scaffolds for promoting bone formation in a critical-size rat calvarial defect model

**DOI:** 10.1080/14686996.2016.1145532

**Published:** 2016-04-08

**Authors:** Haifeng Zhang, Xiyuan Mao, Zijing Du, Wenbo Jiang, Xiuguo Han, Danyang Zhao, Dong Han, Qingfeng Li

**Affiliations:** ^a^ Department of Plastic and Reconstructive Surgery, Shanghai Ninth People’s Hospital, Shanghai Jiao Tong University School of Medicine, No. 639, Zhizaoju Road, Huangpu District, Shanghai, 200011, PRChina; ^b^ Shanghai Key Laboratory of Orthopaedic Implants, Shanghai Ninth People’s Hospital, Shanghai Jiao Tong University School of Medicine, Shanghai, PRChina; ^c^ State Key Lab of Metal Matrix Composites, School of Materials Science and Engineering, Shanghai Jiao Tong University, Shanghai, PRChina; ^d^ Institute of Biomedical Materials, School of Materials Science and Engineering, Shanghai Jiao Tong University, Shanghai, PRChina; ^e^ Shanghai Key Laboratory of Tissue Engineering, Shanghai Ninth People’s Hospital, Shanghai Jiao Tong University School of Medicine, Shanghai, PRChina

**Keywords:** Three-dimensional printing, PLA/HA, β-TCP, DBM, biomaterials, biocompatibility, 30 Bio-inspired and biomedical materials, 102 Porous/Nanoporous/Nanostructured materials, 103 Composites, 211 Scaffold/Tissue engineering/Drug delivery

## Abstract

We have explored the applicability of printed scaffold by comparing osteogenic ability and biodegradation property of three resorbable biomaterials. A polylactic acid/hydroxyapatite (PLA/HA) composite with a pore size of 500 μm and 60% porosity was fabricated by three-dimensional printing. Three-dimensional printed PLA/HA, β-tricalcium phosphate (β-TCP) and partially demineralized bone matrix (DBM) seeded with bone marrow stromal cells (BMSCs) were evaluated by cell adhesion, proliferation, alkaline phosphatase activity and osteogenic gene expression of osteopontin (OPN) and collagen type I (COL-1). Moreover, the biocompatibility, bone repairing capacity and degradation in three different bone substitute materials were estimated using a critical-size rat calvarial defect model *in vivo*. The defects were evaluated by micro-computed tomography and histological analysis at four and eight weeks after surgery, respectively. The results showed that each of the studied scaffolds had its own specific merits and drawbacks. Three-dimensional printed PLA/HA scaffolds possessed good biocompatibility and stimulated BMSC cell proliferation and differentiation to osteogenic cells. The outcomes *in vivo* revealed that 3D printed PLA/HA scaffolds had good osteogenic capability and biodegradation activity with no difference in inflammation reaction. Therefore, 3D printed PLA/HA scaffolds have potential applications in bone tissue engineering and may be used as graft substitutes in reconstructive surgery.

## Graphical abstract

**Figure:**
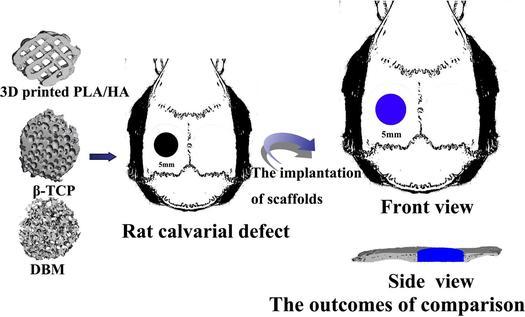

## 1. Introduction

Regeneration of massive bone defects caused by trauma, infection, tumor resection and congenital defects is a major treatment challenge in plastic and reconstructive surgery as well as orthopedic surgery. Various methods have been developed and introduced such as autologous bone grafts, allografts and xenografts in clinic [1]. Although autogenous bone has been widely accepted as the gold standard augmentation material, problems associated with the use include donor site morbidity, limited available bone volume, potential complications and risk [2]. Allografts and xenografts are used for repairing osseous defects with drawbacks in disease transmission and immune rejection [3]. Due to these shortcomings, the researches for different types of bone graft substitute have intensified for decades for filling defects, even when the bone mass is inadequate. [4–6].

The ideal bone substitute materials should fill multiple roles including the ability to deliver cells, support differentiation of regenerative cells and fabricate irregular shapes, biocompatibility, osteoconductivity, osteoinductivity, controlled biodegradability and ultimately the growth of new bone into the augmented area [7–9].

The recent three-dimensional printing technique could provide excellent possibilities for optimizing conventional bone substitutes, with the benefit of controlling the pore interconnection, pore size and overall porosity of the scaffolds [10]. As this new method can print out customized implants with precisely controlled architectures based on three-dimensional computerized tomography or magnetic resonance imaging data files of patients, it has a significant advantage over traditional methods for fabricating three-dimensional porous scaffolds, such as gas foaming, air jet spinning, solvent-casting/salt-leaching and electrospinning techniques [11–14].

Polylactic acid (PLA) and hydroxyapatite (HA) have been approved by the USA Food and Drug Administration (FDA) for biomedical application [15]. PLA is a polymer of lactic acid with high biocompatibility and degradability, and the degradation products of PLA are not toxic [16]. PLA is fabricated into porous scaffolds, which provide impetus for bone repair by supplying a space for bone cell growth and differentiation both *in vitro* and *in vivo* [17]. However, the weak mechanical properties and inflammatory and allergic reactions of PLA limit applications [18]. HA has been widely used to prepare composite materials as the biomineral in bone repair. HA acts as a reinforcing material to improve the mechanical properties of polymers and improves the bioactivity and the osteoconductivity of the scaffold [19]. PLA and HA are mixed to compose a porous scaffold that the behavior of ceramic (HA) in the polymeric matrix has a dose dependent effect, such as mechanical behavior, degradation rate, and osteoconduction [20, 21].

Beta-tricalcium phosphate (β-TCP) ceramics have been extensively applied in animal experiments and clinical studies [22, 23]. Structurally similar to cancellous bone, β-TCP ceramics are biodegradable inorganic bone substitutes with inorganic components and have been used as bone-filling materials for cell migration and rapid bone formation due to their osteoconductivity and bone replacement capability [24].

Demineralized bone matrix (DBM) is an acid-extracted organic matrix derived from bone sources and is an osteoconductive and osteoinductive biomaterial due to its content of osteogenic factors, including bone morphogenetic proteins (BMPs) and other osteogenic non-collagenous proteins [25, 26]. Partially demineralized biomaterial is a kind of DBM, in contrast to fully demineralized matrix, partially DBM had the advantage of providing immediate structural support to unstable osseous defects [27–29].

As bone repair is one of the targeted applications for the implants described here, they were tested preclinically in a cranial model. The critical size defect model (CSD) is often used to study materials, as it eliminates the effects of motion at the defect site and facilitates the operation and the analysis as well as need not implant fixation [30]. Different types of bone graft substitutes could be used to fill a CSD. However, there has been no comprehensive study to evaluate and compare the major categories of resorbable materials. The objectives of the present study were to fabricate PLA/HA composite scaffolds by 3D printing and evaluate bone repairing capacity and inflammation *in vivo* as well as to compare the effects of inorganic and organic elements of bone on the proliferation and osteogenic differentiation *in vitro* using three-dimensional β-TCP ceramics and partially DBM.

## 2. Materials and methods

### 2.1. Fabrication of the biomaterials

**Figure 1.**
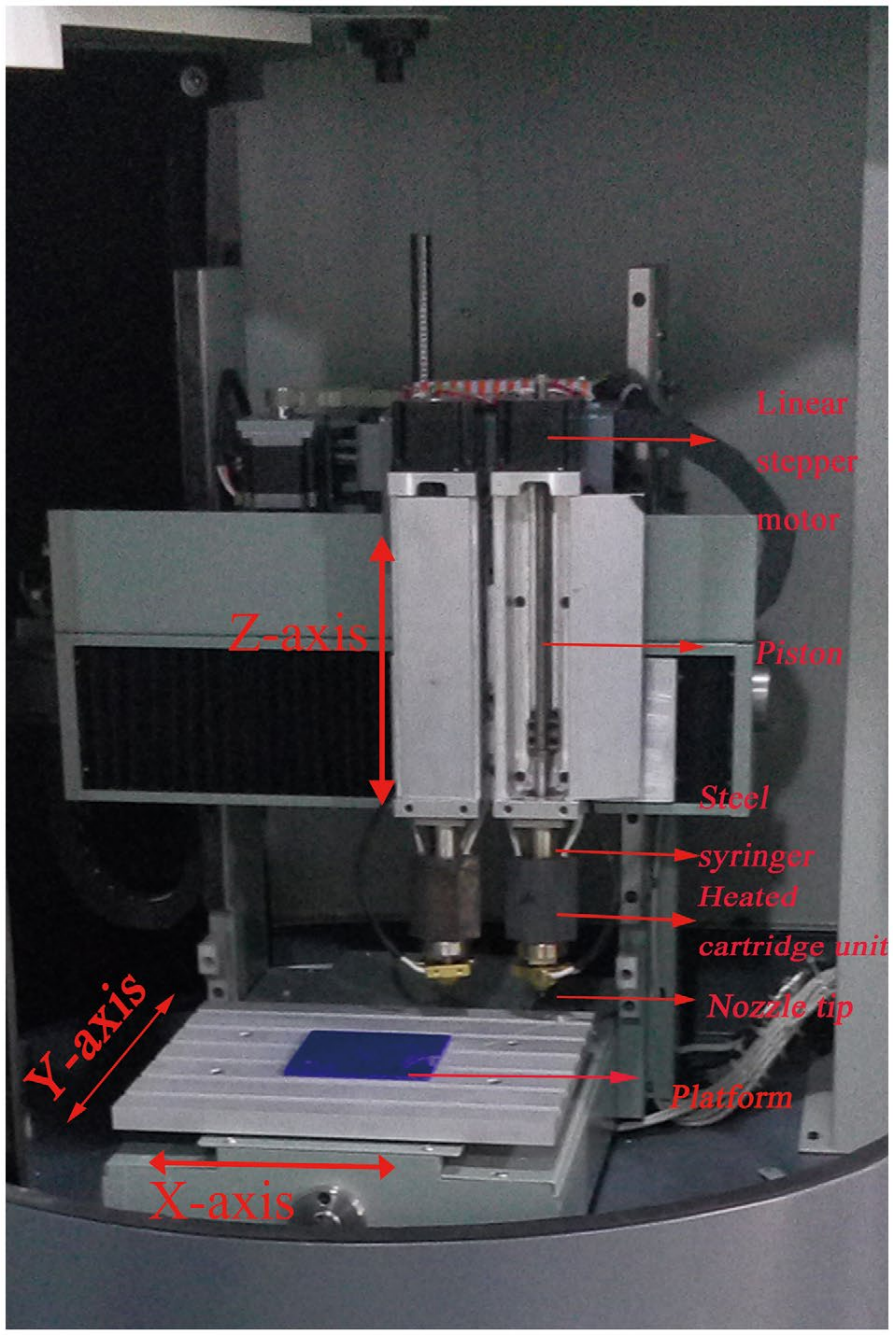
3D printing machine using the new mini-deposition system.

Poly (L-lactide) (PLA) was purchased from Sigma-Aldrich (Shanghai, China) and hydroxyapatite (Ca _10_(PO_4_)_6_(OH)_2_) (HA) was purchased from Sonac Company (Tauranga, the Netherlands) with the mean size of 2.1 ± 0.4 μm. Unlike the conventional FDM 3D printing, a new mini-deposition system (MDS) located in Shanghai 3D printing center was developed to fabricate scaffolds (Figure 1). PLA (85 wt%) and HA (15 wt%) were mixed and processed as the raw materials to produce PLA/HA composite scaffolds with the MDS method [31, 32]. Based on three-dimensional computed tomography reconstruction and computer-aided-design/computer-aided-manufacturing system, the 3D printer produced porous cylindrical scaffolds with predetermined structure (5 mm diameter, 2 mm height). The 3D printed scaffolds were sterilized by ethylene oxide and prepared for use.

The β-TCP ceramic scaffolds with a mean pore size of 400 μm and porosity > 50% were provided by Dr J. X. Lu (Institut de Recherche sur les Maladies du Squelette, Université du Littoral Côte d’Opale, Berck sur Mer, France) and were sterilized by dry heat at 180°C for 1 h [33].

The partially DBM scaffolds were prepared from the cancellous bone, harvested from the proximal femurs of four commercially obtained porcine bone (Shanghai, China). The samples were cleaned of residual tissue, sectioned into cylinders (5 mm diameter, 2 mm height) and stored at -20°C to preserve their osteoinductivity until further preparation [28] The partially DBM scaffolds were prepared by a modification of methods as previously reported [26, 27]. In order to facilitate residual lipid extraction, samples were incubated in anhydrous ethyl ether for 12 h and then the samples were partially demineralized in 0.6 N HCl for 15 min (50 ml g^–1^ bone), and rinsed in distilled water for 2 h to neutralize the pH. In order to further increase group homogeneity, 5 mg sections from all samples were assayed for calcium content as described by Liu et al. [27]. The scaffolds were sterilized by ethylene oxide and stored at 4°C until use.

### 2.2. *In vitro* cellular responses of BMSCs to three scaffolds

#### 2.2.1. Cell culture

BMSCs were harvested from adult male Sprague–Dawley (SD) rats. The femurs and tibia were aseptically dissected, followed by condyles resection using a site cutter and the bone marrow was flushed with Dulbecco minimum essential medium (DMEM; Gibco, Australia) supplemented with 10% fetal bovine serum (FBS; Hyclone; Logan, Utah, USA), penicillin (50 U ml^–1^) and streptomycin (50 mg ml^–1^). The cell pellet was re-suspended in DMEM medium and directly transferred to a 10 cm culture dish. Medium was exchanged twice a week.

#### 2.2.2. Cell attachment and proliferation

All the three kinds of scaffold were sterilized in an ethylene oxide sterilizer for 12 h, then transferred into 24-well cell culture plates and incubated with culture medium for 4 h prior to cell seeding in a humidified atmosphere of 5% CO_2_.

In order to assess cell attachment on three-dimensional printed (3DP) PLA/HA, β-TCP and DBM scaffolds, 1 × 10^5^ bone marrow stromal cells were seeded on each scaffold in a 24-well plate and allowed to adhere to the scaffold for 3 h. Subsequently, the cells were incubated in DMEM medium supplemented with 10% FBS and 1% penicillin/streptomycin in humidified culture conditions. The samples were removed from the culture wells, rinsed with phosphate buffered saline (PBS), and the relative adhesion rate of BMSC cultured on the three scaffolds was estimated using the Cell Counting Kit-8 assay (CCK-8; Dojindo Molecular Technologies Inc., Kumamoto, Japan) for 4, 8 and 12 h. The relative adhesion rate was measured as the ratio of optical densities (OD) of cells in scaffolds and total cells.

Adhesion of BMSCs to the biomaterials was assessed by scanning electron microscopy (SEM, FEI Quanta 250, Hillsboro, Oregon, USA) after seven days. Biomaterials seeded with cells were fixed for 1 h with 2.5% glutardialdehyde and subsequently dehydrated for 15 min in graded ethanol (50, 70, 90, 95, 100%) and hexamethyldisilizane. The samples were sputtered with gold and the morphological characteristics of the attached cells were observed using SEM.

The proliferation of BMSCs cultured on all scaffolds was measured using CCK-8. Initially, BMSCs were cultured on scaffolds and without scaffolds at a density of 1 × 10^4^ cells for one, four and seven days. Subsequently, 450 μl of culture medium and 50 μl CCK-8 solution were added to each well at each time point and incubated at 37°C for another 4 h. An aliquot of 100 μl was sucked out from each well and transferred to a fresh 96-well plate. The light absorbance of these samples was measured at 450 nm with a microplate reader (Infinite M200PRO TECAN, Tecan Group Ltd., Männedorf, Switzerland). The absorbance of blank wells was subtracted from all results.

To observe the cell proliferation state, BMSCs with lentiviral transduction were incubated with three kinds of scaffolds. Rat BMSCs were transfected with Lenti-Enhanced Green Fluorescent Protein (EGFP) as described by Naldini [34] and Zhu [35]. Rat BMSCs at passage 2 were infected with Lenti-EGFP in the presence of 5 μg ml^–1^ polybrene at a multiplicity of infection (MOI) of 10. Before seeding the cells, three kinds of scaffold were pre-immersed in DMEM with 10% FBS for 24 h. Green fluorescent protein (GFP)-positive cells were collected and resuspended to a concentration of 2×10^7^ cells ml^–1^.

A total of 20 μl cell suspension was seeded on each piece of scaffold in 24-well plates and 500 μl of complete medium was added to each well (*n*=6) after two hours. The survival state of the GFP+cells on the scaffolds was observed by fluorescence microscopy at two, seven and 14 days, respectively.

#### 2.2.3. Alkaline phosphatase (ALP) activity and osteogenesis related gene expression

To assess ALP activity of BMSCs cells grown on all scaffolds, 1×10^5^ cells were seeded on each scaffold and cultured in 24-well plates for three, seven and 14 days. At the predetermined time point, culture medium was decanted and the cell layer washed gently three times with PBS followed by washing once in cold 50 mM Tris buffer, and then cells were lyzed in 200 μl of 0.2% Triton X-100. Lysates were sonicated after being centrifuged at 10,000 rpm for 5 min at 4°C. 50 μl of supernatant was mixed with 150 μl working solution according to alkaline phosphatase assay kit (Sigma). The conversion of p-nitrophenylphosphate into p-nitrophenol in the presence of ALP was determined by measuring the absorbance at 520 nm with a microplate reader (Infinite M200PRO TECAN). The ALP activity was calculated from a standard curve after normalizing to the total protein content, which was determined by a Bio-Rad DCTM protein assay kit (Bio-Rad) at 690 nm with a microplate reader (Infinite M200PRO TECAN). The results were expressed in lM of p-nitrophenol produced per min per mg of protein.

The quantitative real-time reverse transcription polymerase chain reaction (qRT-PCR) method was used to measure the osteogenic differentiation of BMSCs on three scaffolds. Osteogenic related genes (osteopontin (OPN) and collagen type I (COL-1)) were selected for the estimation, and compared in three materials. The 1×10^5^ cells were seeded per scaffold and cultured for seven and 14 days in culture medium. The total cellular RNA on the scaffold was extracted with TRIzol reagent (Invitrogen Pty Ltd, Victoria, Australia) and reverse-transcribed into cDNA using with PrimeScript^TM^ RT reagent Kit (Takara, Tokyo, Japan) and the qRT-PCR analysis was performed on an 7500 Real Time PCR System (Applied Biosystems, Foster City, CA, USA) using SYBR Green detection reagent. The relative expression of the genes of interest was normalized against the housekeeping gene glyceraldehydes 3-phosphate dehydrogenase (GAPDH) [36]. The mean cycle threshold (Ct) value of each target gene was normalized against the Ct value of GAPDH. The relative expression level for each target gene was evaluated using the 2^-(normalized average Ct)^ ×10 method [37].

### 2.3. *In vivo* study of critical-sized rat calvarial defect model

#### 2.3.1. Ethics

Procedures involving animals were conducted in accordance with the guidelines of the Institutional Animal Experiment Department of Shanghai Jiao Tong University (Shanghai, China; Animal Ethics Approval #201040) and principles of laboratory animal care (NIH publication number 85–23, revised 1985) and ethics committee specifically approved this study.

#### 2.3.2 Surgical procedure and treatment

All surgical procedures were performed on eight-week old male Sprague–Dawley rats, weighing approximately 300–350 g and all rats acclimated for 14 days prior to surgery. The animals were anesthetized with 5% ketamine. Following anesthesia, the surgical areas were shaved and disinfected with povidone-iodine. A 1.0–1.5 cm vertical incision was made on the proximal-medial area of the calvaria and the soft tissue and the periosteum were elevated to expose calvaria. Unilateral critical-sized defect was created in each rat with micro drills (Stryker, Mahwah, NJ, USA) of 5 mm in diameter at low rotation speed with constant irrigation. The 32 rats were randomly allocated to the following graft study groups: (1) 3DP PLA/HA (*n* = 8), (2) β-TCP (*n* = 8), (3) DBM (*n* = 8) and (4) blank control (*n* = 8). The periosteum was repositioned and sutured with a 5–0 PDS suture. Then the skin was sutured by 4–0 silk suture. Following the operation, the animals were allowed rat food and water ad libitum. After four or eight weeks, the animals were sacrificed with carbon dioxide gas suffocation.

#### 2.3.3. Blood collection and hematological analysis

In order to assess local inflammatory reaction after implanting different kinds of materials, blood samples were collected and analyzed with a routine blood test. At 12 days, four weeks, six weeks and eight weeks after surgery, blood was sampled from heart puncture in tubes containing ethylenediaminetetraacetic acid (EDTA). With the help of a counting chamber, total leukocyte and red cells numbers as well as hemoglobin content were assessed. Differential counts of white blood cells including lymphocytes, monocytes, eosinophils, and basophils were determined

#### 2.3.4. Micro-computed tomography analysis (μCT)

After sacrificing the animals at four and eight weeks post-operatively, the skulls were dissected out and fixed in neutral 10% formalin then examined with micro-CT (Scanco Medical AG, Basserdorf, Switzerland). Besides, micro-CT scanned at 10 μm resolution for undecalcified tissue samples and original materials that were used to evaluate new bone formation and materials degradation rates. Three-dimensional images were reconstructed using 3D Creator software. Three-dimensional measurements of the amount of bone volume per total volume (BV/TV) and residual materials volume fraction (RMvF) [38] in the bone defect were also calculated using the analysis software.

#### 2.3.5. Histological observation

Subsequent to μCT analysis, the specimens were decalcified by immersing in 10% EDTA for four weeks at 37°C and during this period the solution was changed three times.

Decalcified specimens were dehydrated in ascending grades of ethanol and embedded in paraffin. To compare and visualize new bone formation, 5 μm thick serial slices with a microtome were sectioned starting from the middle region of the defect area. Sections in all groups were stained with hematoxylin and eosin (HE). Immunohistochemical staining was further carried out to assess and analyze the formation of new bone. Sections were then incubated with the primary antibody: against osteocalcin (OC) and type I collagen (COL-1) (Abcam, Cambridge, MA, USA) at 4°C overnight. After rinsing with the PBS buffer, the HRP-conjugated secondary antibody and 3,3′-diaminobenzidine (DAB) solution was used to stain the sections, and then cell nuclei were to identified with hematoxylin. Immunohistochemical experiment was performed on four samples per group (*n* = 4). The images were acquired using a microscope (Leica DM 4000B, Wetzlar, Germany) with BioQuant OSTEO II software (BioQuant Image Analysis Corporation, Nashville, TN, USA). The OC and COL-1 positive areas were calculated using Image-Pro Plus 6.0 software (SPSS 17.0, Chicago, IL, USA) to evaluate the formation of new bone.

### 2.3.6. Statistical analysis

The data were presented as mean ± standard deviation. The statistical significances of the differences among the various groups were examined with one-way ANOVA followed by the least significant difference (LSD) test. SPSS 17.0 was employed for all statistical analyses and *p-*values less than 0.05 were considered to be significant.

## 3. Results

### 3.1. Characterization of scaffolds

**Figure 2.**
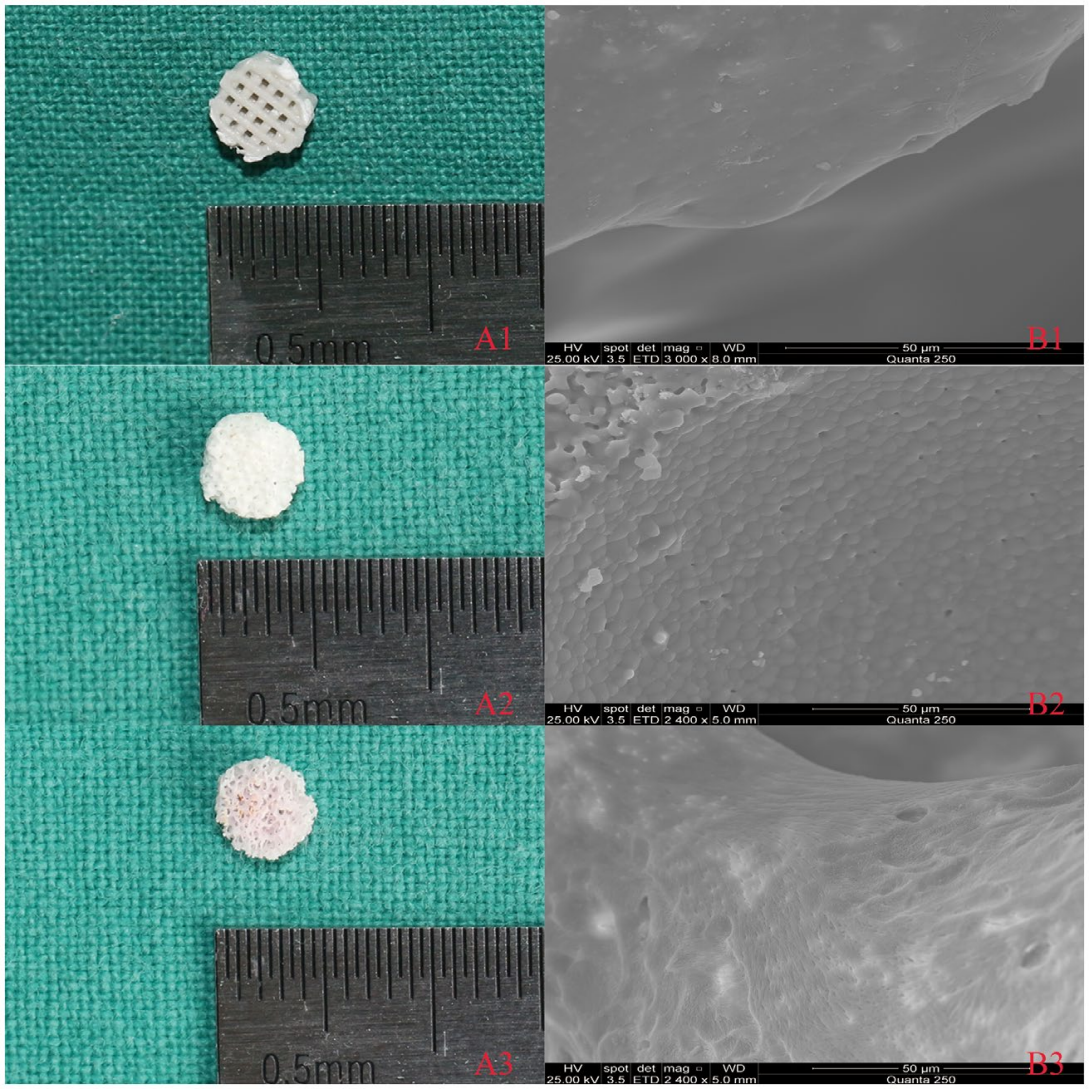
(A) Optical and (B) SEM images for three scaffolds: 3D printed PLA/HA (1), β-TCP (2) and DBM (3).

The cylindrical structure was recorded with the size of 5 mm×2 mm and surface morphology of the scaffolds was detected by scanning electron microscopy (Figure 2). All three types of material had relatively large pore sizes and similar porosity (Table 1). The scaffolds of 3D printed PLA/HA (85 wt%: 15% wt%) had relatively compact porosity with an average value of 60%; the mean pore size was 500 μm, as expected. β-TCP ceramic scaffolds had a mean porosity of 60% and a pore diameter of 50–600 μm. The mean porosity and pore size of partially DBM were 62% and 520 μm, respectively. The calcium content of partially DBM from porcine femurs was 15.30 ± 0.95%.

**Table 1.** Scaffold properties.

Indices	3DP PLA/HA	β-TCP	DBM
Porosity (%)	60.0 ± 1.5	60 ± 10	62 ± 4
Pore size (μm)	500 ± 20	400 ± 247	520 ± 290

### 3.2. *In vitro* cellular responses to scaffolds

#### 3.2.1. Growth and proliferation of BMSCs on scaffolds

**Figure 3.**
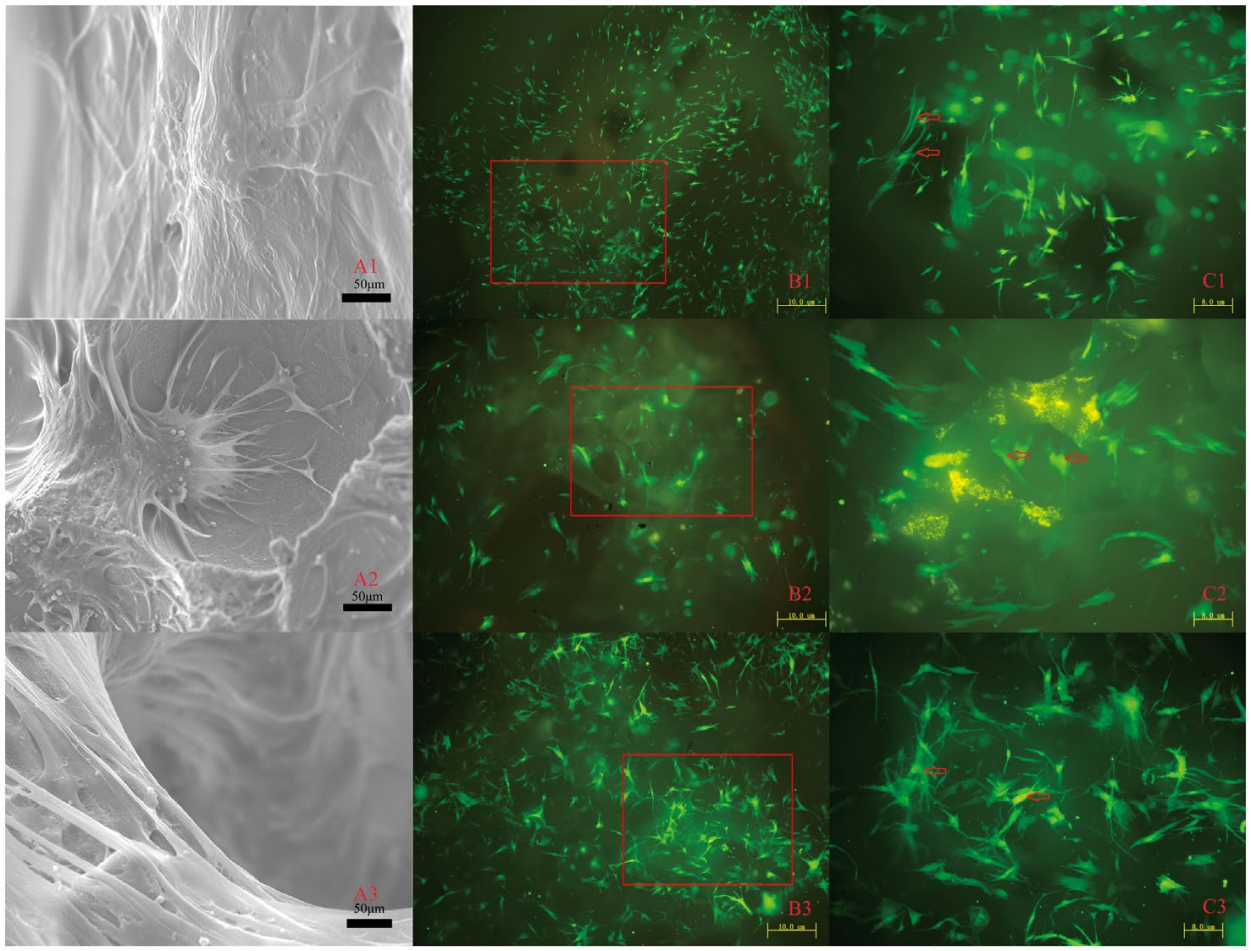
BMSC responses to three scaffolds. (A) SEM images of the attachment of BMSCs on (1) PLA/HA, (2) β-TCP and (3) DBM scaffolds after culturing for seven days. (B, C) The fluorescence images of proliferation of BMSCs on (1) PLA/HA, (2) β-TCP and (3) DBM scaffolds after culturing for seven days. (← The state of proliferation of BMSCs; ^▄^ the selected areas in B corresponding to C. Length scales: (A) 50 μm, (B) 10 μm and (C) 8 μm).

**Figure 4.**
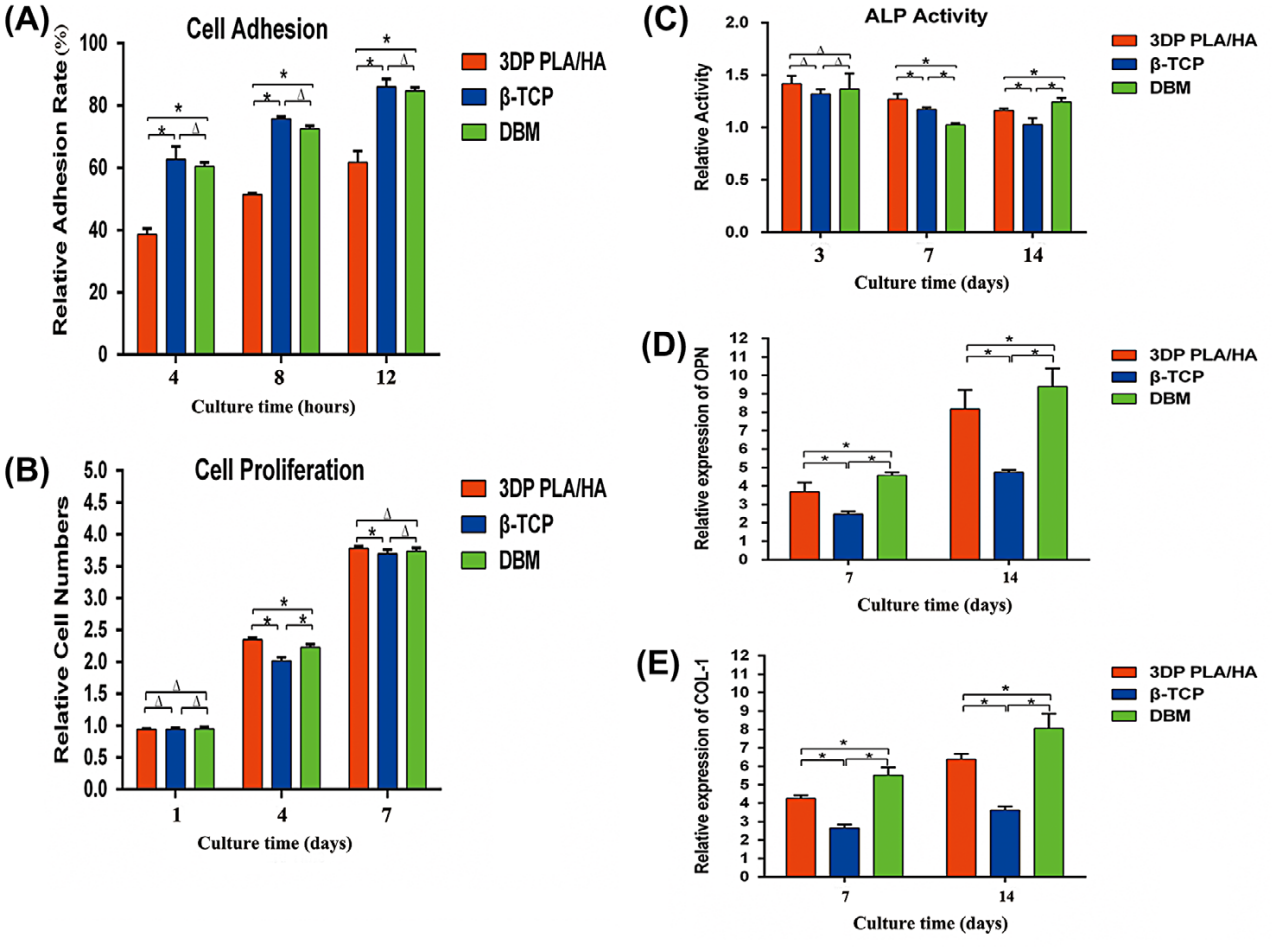
Quantitative analysis of three scaffolds: (A) cell adhesion rate, (B) cell proliferation, (C) ALP activity, (D) osteogenic gene expression and (E) COL-1 of BMSCs cultured on different scaffolds (*p＜0.05).

To visualize cell growth and spatial distribution of the three scaffolds, BMSCs were used in the study. After seven days of culture, the attachment and morphology of BMSCs were examined by SEM. BMSCs were attached to the surface of the pore struts, presenting well-stretched morphology on each type of scaffold (Figure 3(A)). The cell adhesion ability results for the three samples were determined by the measured the relative cell adhesion rate at 4, 8 and 12 h. The quantitative data demonstrated that there are significantly more adhesive cells on the β-TCP and DBM than on the 3DP PLA/HA specimen (Figure 4(A)). However, a number of cells remained attached to 3DP PLA/HA scaffolds with the cell adhesion rate over 60%.

**Figure 5.**
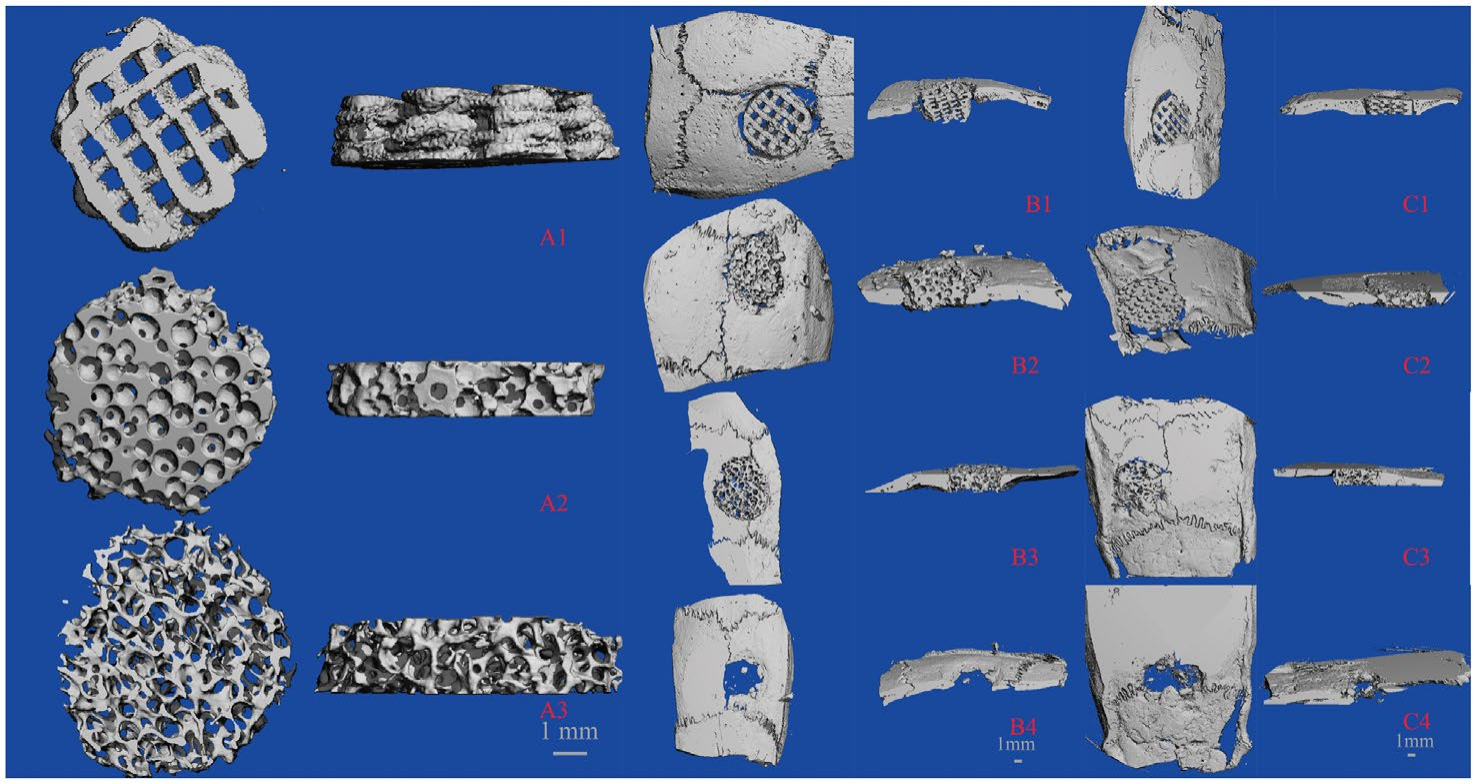
Micro CT images for three scaffolds. (A) Plane and profile images of (1) PLA/HA, (2) β-TCP and (3) DBM scaffolds before implanting. (B) Typical micro-CT images of (1) PLA/HA, (2) β-TCP and (3) DBM scaffolds as well as (4) bone defect without scaffold at four weeks *in vivo*. (C) Micro-CT images of (1) PLA/HA, (2) β-TCP and (3) DBM scaffolds as well as (4) bone defect without scaffold at eight weeks *in vivo*.

The proliferation of BMSCs cultured on three kinds of scaffolds were observed for one, four and seven days. As shown in Figure, the GFP positive BMSCs exhibited good viability and compatibility with all scaffolds and could remain stable over the entire culture period (Figure 3(B) and (C)). To estimate the relative cell number at different time points, CCK-8 proliferation assay was used. There are no statistically significant differences observed among the three samples at day 1. However, at day 4, the cell numbers on the 3DP PLA/HA scaffolds were significantly higher than those on the β-TCP and DBM scaffolds (*p* < 0.05) (Figure 4(B)).

#### 3.2.2. Osteogenesis of BMSCs on scaffolds

To explore whether 3DP PLA/HA supported the osteogenic potential of BMSCs on the scaffolds, ALP activity of BMSCs cultured on three types scaffolds for three, seven and 14 days were measured. As shown in Figure 4(C), no significant differences could be found between these three groups at day 3, while the 3DP PLA/HA scaffolds exhibited a significant enhanced ALP activity at days 7 and 14 compared to the other scaffolds (*p* < 0.05).

Cell differentiation of BMSCs on all scaffolds was further analyzed by osteogenic-related genes determined by the expressions of osteogenic markers OPN and CoL-1 at seven and 14 days. The secretion of OPN and CoL-1 in these three groups kept increasing after seeding, and no decline was found within 14 days. The levels of expression of osteogenic-related genes was upregulated on 3DP PLA/HA scaffolds compared to β-TCP scaffolds (*p* < 0.05) and lower than DBM scaffolds (*p* < 0.05) at both seven and 14 days, which indicated the 3DP PLA/HA scaffolds could promote osteogenic differentiation to some extent (Figure 4(D) and (E)).

### 3.3. Bone regeneration and inflammatory response in critical-sized rat calvarial defects

#### 3.3.1. Hematology analysis

Differential leukocyte cell counts and red blood cell (RBC) levels were similar in all implanted groups at the four time points (12 days, and four, six and eight weeks after the surgery) and hemoglobin was maintained at a normal level at all the time points (Table 2). No relevant differences between the implanted groups and nontreated groups were found for any hematological parameter at eight weeks. No significant differences between the groups were apparent for the red blood cell count and hemoglobin, whereas the white blood cell counts were slightly decreased in the DBM group, when compared to the long-term inflammatory reactions in the control group.

**Table 2.** Hematological variables in blood from rats at different times after surgery.

Duration of implantation	Rat type	WBC (×10^**3**^ mm^–**3**^）	Lymphocytes (%)	Monocytes (%)	Eosinophilics (%)	Basophils (%)	RBC (×10^**6**^ mm^–**3**^）	Hb (×g dl^–1^)
12 days	3DP PLA/HA	13 (±2)	80 (±2)	3 (±1)	0 (±0)	0 (±0)	7.9 (±0.2)	15.8 (±0.5)
	β-TCP	13 (±3)	82 (±3)	5 (±3)	2 (±1)	0 (±0)	7.7 (±0.2)	15.2 (±0.2)
	DBM	12 (±2)	87 (±4)	3 (±1)	2 (±0)	0 (±0)	9.1 (±0.3)	15.5 (±0.6)
	Nontreated	13 (±2)	83 (±2)	3 (±2)	1 (±0)	0 (±0)	7.0 (±0.2)	14.1 (±0.3)
Four weeks	3DP PLA/HA	13 (±3)	81 (±2)	5 (±3)	1 (±0)	0 (±0)	8.8 (±0.1)	16.2 (±0.8)
	β-TCP	13 (±4)	83 (±3)	5 (±3)	0 (±0)	0 (±0)	9.1 (±0.3)	17.7 (±0.5)
	DBM	10 (±2)	81 (±2)	4 (±1)	2 (±1)	0 (±0)	9.1 (±0.2)	16.7 (±0.3)
	Nontreated	12 (±3)	80 (±2)	4 (±1)	0 (±0)	0 (±0)	8.9 (±0.2)	17.5 (±0.6)
Six weeks	3DP PLA/HA	12 (±3)	85 (±4)	4 (±1)	2 (±1)	0 (±0)	7.9 (±0.1)	14.9 (±0.2)
	β-TCP	11 (±2)	86 (±2)	6 (±3)	2 (±2)	0 (±0)	8.1 (±0.2)	14.2 (±0.2)
	DBM	11 (±1)	85 (±3)	4 (±2)	1 (±1)	0 (±0)	7.8 (±0.1)	15.3 (±0.3)
	Nontreated	11 (±2)	84 (±3)	4 (±2)	2 (±1)	0 (±0)	9.1 (±0.3)	16.8 (±0.5)
Eight weeks	3DP PLA/HA	12 (±3)	82 (±2)	3 (±1)	2 (±1)	0 (±0)	8.1 (±0.2)	15.0 (±0.2)
	β-TCP	11 (±2)	80 (±2)	3 (±2)	2 (±2)	0 (±0)	8.3 (±0.2)	14.4 (±0.3)
	DBM	8 (±1)	84 (±3)	4 (±3)	1 (±1)	0 (±0)	8.1 (±0.2)	13.7 (±0.2)
	Nontreated	12 (±2)	81 (±2)	4 (±2)	1 (±1)	0 (±0)	9.1 (±0.3)	15.8 (±0.4)

Abbreviations: WBC, white blood cells; Hb, hemoglobin.

#### 3.3.2. Micro-CT measurement

**Figure 6.**
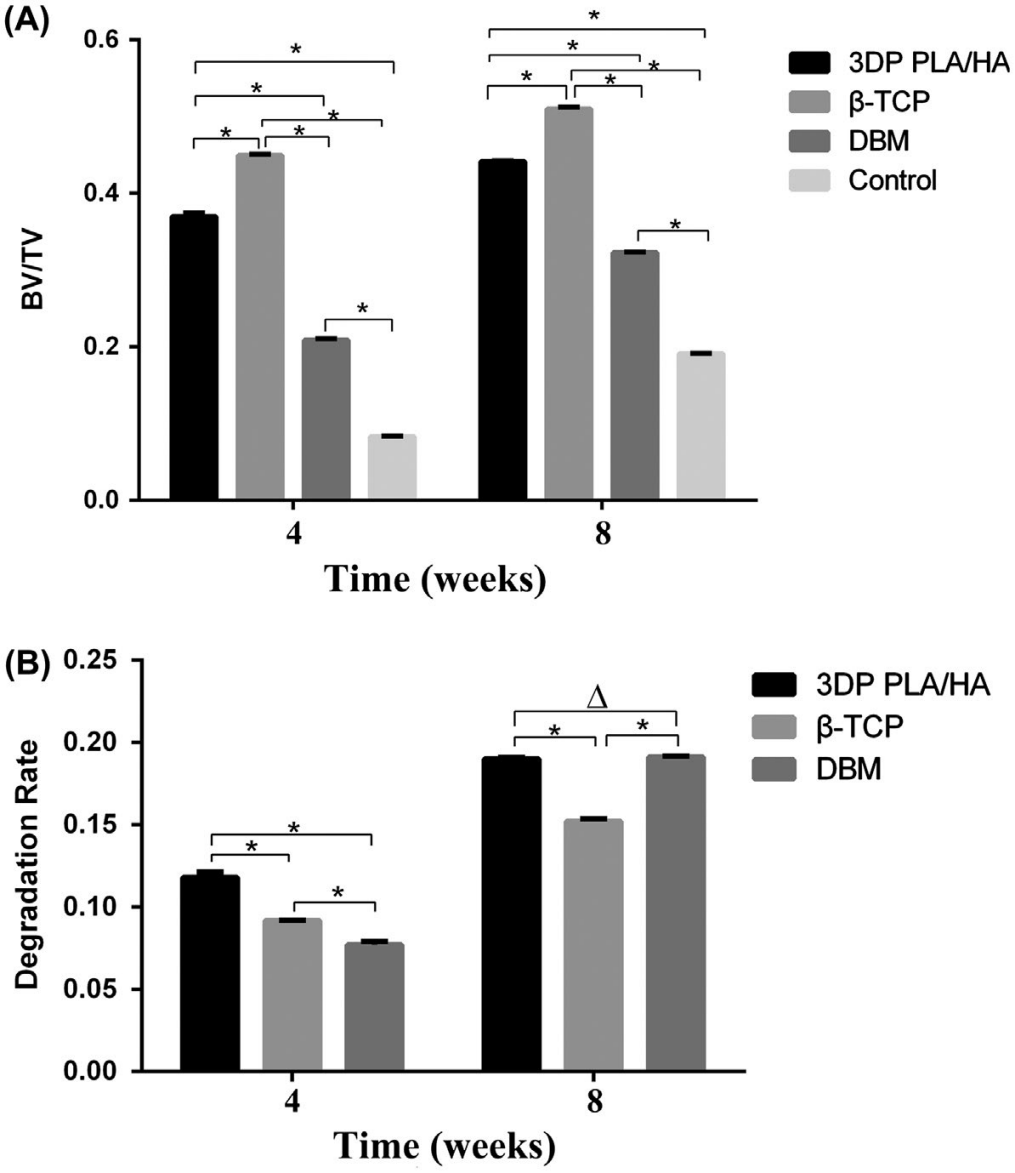
BV/TV and degradation rate in every group (A) Percentage of new bone formation at four and eight weeks. (B) Percentage of degradation of three scaffolds at four and eight weeks. (*p＜0.05)

The morphology of the newly formed bone was reconstructed by micro-CT (Figure 5). The results indicated that the relative newly formed bone area (BV/TV) in 3DP PLA/HA scaffolds was greater than that in DBM scaffolds and lower than the group β-TCP at four weeks after implantation (Figure 6(A)). Similarly, at eight weeks after implantation, the same tendencies were observed and the BV/TV value at eight weeks after implantation was higher than those at four weeks after implantation. In defects without any implants, all new bone areas were less than other implanted group.

In order to study the correlation relationship between the degradation rate of scaffolds and the growth rate of newly formed bone, the volume fraction of residual materials was compared and analyzed. As seen from the results in Figure 6(B), there was a significant difference in the degradability of the three bone substitute scaffolds. At four weeks after implantation, the degradation rates in 3DP PLA/HA scaffolds were higher than the other two materials and the group in DBM showed slower resorption than the β-TCP groups. Interestingly, the results of total degradation rates showed no significant difference between 3DP PLA/HA scaffolds and DBM scaffolds at eight weeks and β-TCP had the lowest degradation rates in all groups.

#### 3.3.3. Histological examination of new bone formation

**Figure 7.**
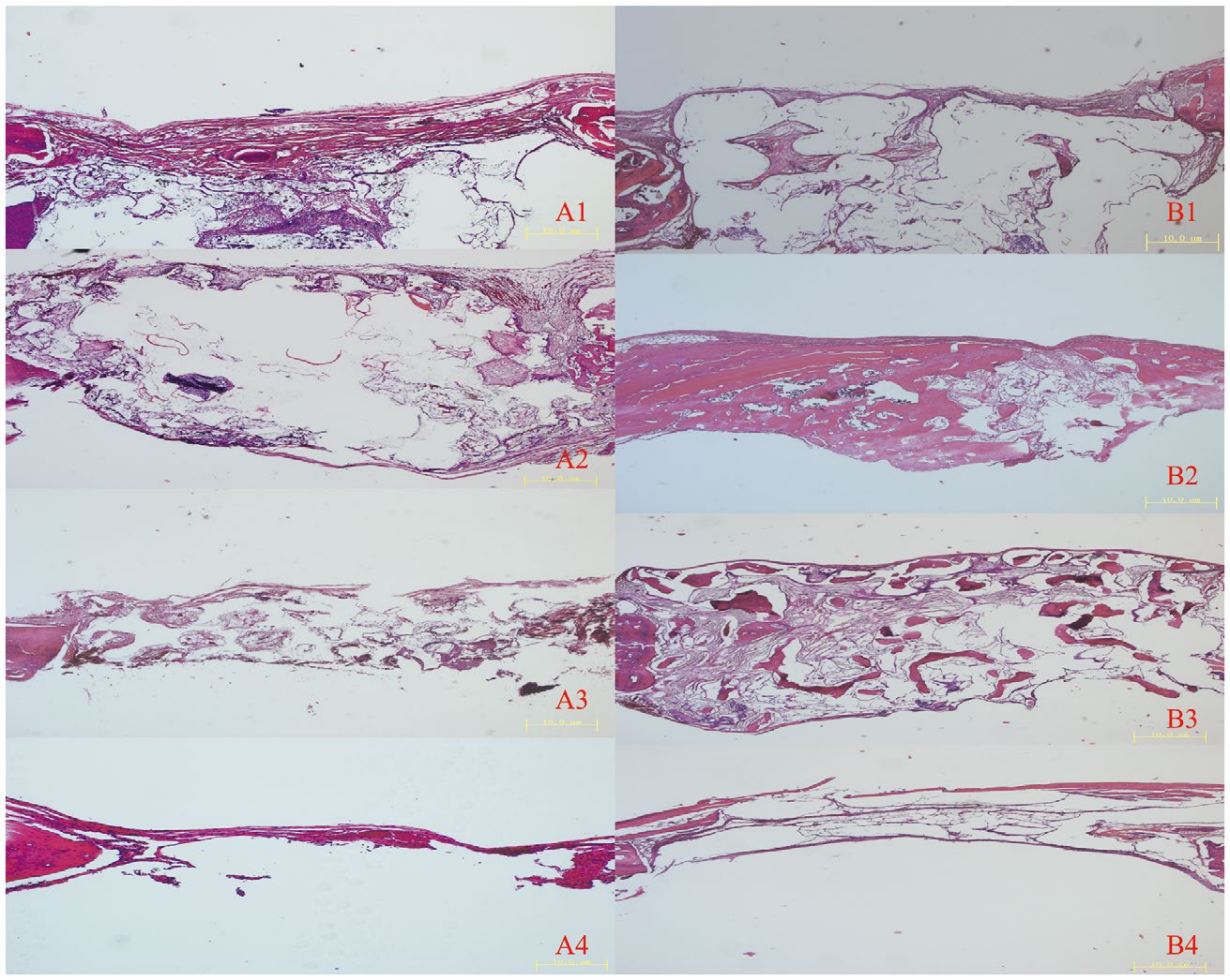
HE images of implanted and control group after four and eight weeks. (A) Histological images of implanted (1) PLA/HA, (2) β-TCP and (3) DBM scaffolds as well as (4) control group four weeks after implantation. (B) Histological images of (1) implanted PLA/HA, (2) β-TCP and (3) DBM scaffolds as well as (4) control group eight weeks after implantation. Scale bars 10 μm.

Analysis of decalcified specimens stained with HE showed that newly formed bone was observed in the implanted group compared to the control group. The newly formed bone was identified around and in close contact with the materials at four and eight weeks after implantation (Figure 7). However, the new bone failed to progress toward the center of the defect and the central portion of defect was filled with compressed fibrous connective tissue in the non-treated group.

**Figure 8.**
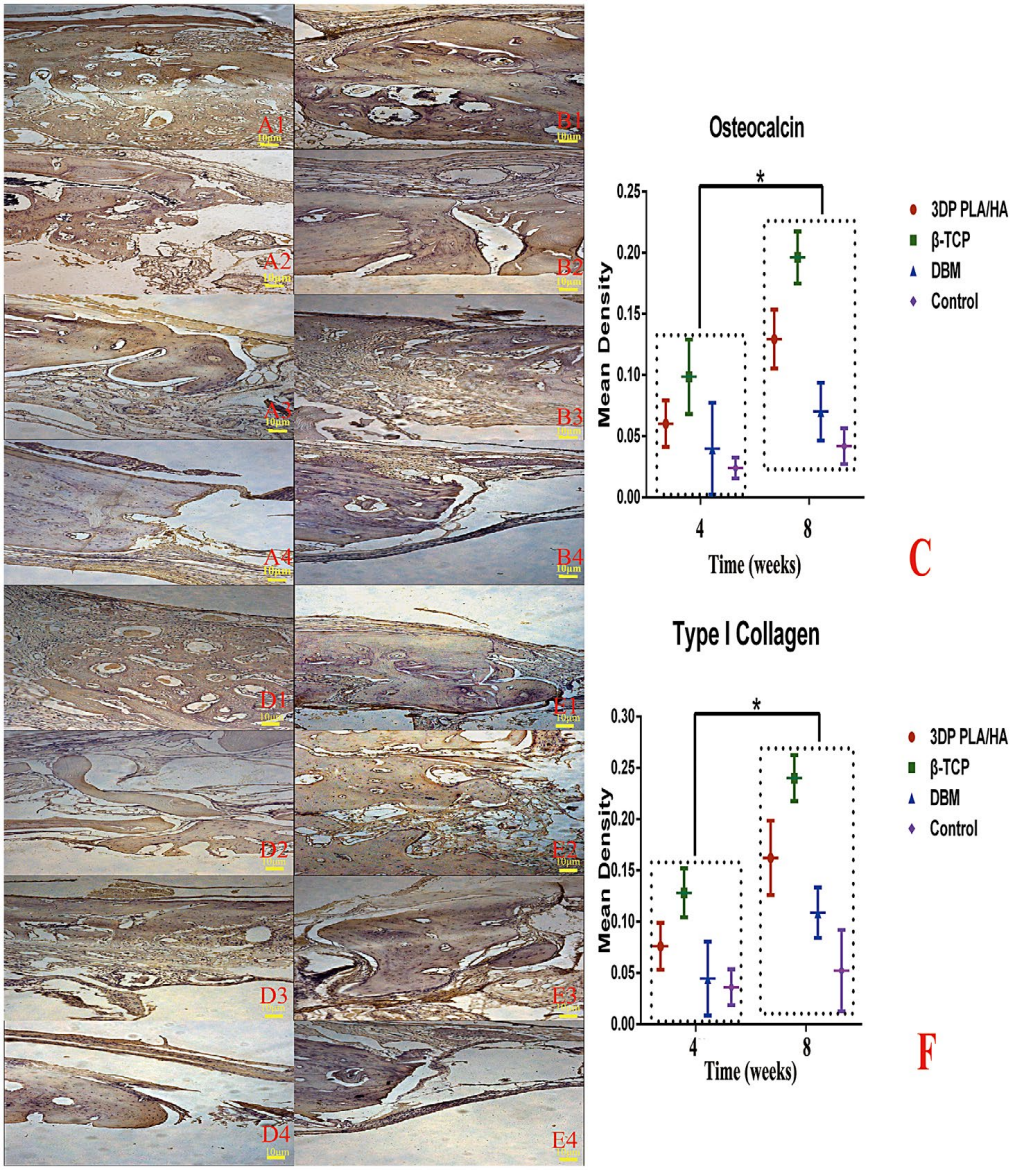
Immunohistochemical examination of osteocalcin and type I collagen were carried out in the margin of bone defect in different time (magnification ×100). (A) Osteocalcin expression images of implanted (1) PLA/HA, (2) β-TCP and (3) DBM scaffolds as well as (4) control group four weeks after implanting. (B) Osteocalcin expression images of implanted (1) PLA/HA, (2) β-TCP and (3) DBM scaffolds as well as (4) control group eight weeks after implanting. (C) Semi-quantitative scatter plot of osteocalcin expression. (D) Type I collagen expression images of implanted (1) PLA/HA, (2) β-TCP and (3) DBM scaffolds as well as (4) control group four weeks after implanting. (E) Type I collagen expression images of implanted (1) PLA/HA, (2) β-TCP and (3) DBM scaffolds as well as (4) control group eight weeks after implanting. (F) Semi-quantitative scatter plot of type I collagen expression (*p＜0.05).

In relation to bone regeneration, newly formed bone cell surface markers OC and COL-1 were assessed by immunohistochemistry in each group. As shown in Figure 8, a few newly formed bone tissues were detected in the non-treated group; by comparison, more positive expression of OC and COL-1 were detected in the 3DP PLA/HA group than the DBM group, which was less than that of β-TCP group (p < 0.05). The results indicated that the percentage of new bone area in 3DP PLA/HA scaffolds was larger than in DBM scaffolds and control groups, but less than the β-TCP group. This suggested 3DP PLA/HA scaffolds could enhance bone regeneration capacity after eight weeks of implantation in rats.

## 4. Discussion

Over the last decade, bone graft substitutes in bone engineered tissues have been developed as alternatives to autografts and allografts to repair and reconstruct bone defects [39]. Bone grafts involve resorbable, non-resorbable and minimally resorbable biomaterials used alone or combined with other materials [40]. According to their ability to allow or induce bone formation, resorbable substitutes can further be divided into osteoconductive and osteoinductive materials [41, 42]. Many studies have compared different kinds of resorbable scaffold with various traditional fabrication techniques [42–44] and those materials meet earlier requirements. However, comparisons of preformed resorbable matrices with the use of modern 3D printing techniques and other traditional resorbable scaffolds have seldom been reported.

Materials that are designed to mimic the three-dimensional characteristics of autograft tissue have their advantages and limitations. As for polymer-ceramic composites, 3DP PLA/HA could own the advantages in two kinds of scaffolds, such as degradation time, biocompatibility and osteoconductivity as well as ease of manufacture into different physical structures [45, 46]. However, scaffolds made of polymers and the degraded acidic monomers cause inflammation during dissolution [47], and due to the relative smooth surface of the material, this property would affect cells attach and behavior [45].

β-tricalcium phosphate (β-TCP) ceramics closely mimic bone tissues and exhibit bioresorbability and osteoconductivity [48, 49]. While undergoing degradation processes, β-TCP scaffolds have a volume instability that does not allow new bone formation to retain the original volume [50].

DBM, an allograft material, is an osteoconductive and osteoinductive commercial biomaterial [25]. The osteoinductivity of DBM could be attributed to osteoinductive growth factors that are buried within the mineralized matrix and enhance the bone formation. In contrast to fully DBM, partially DBM has the merit of maintaining mechanical strength and providing immediate structural support to unstable osseous defects [28]. Although it has a long record of clinical use, many patients are reluctant to accept allograft materials [51].

In an *in vitro* study, the adhesion, proliferation, ALP activity and osteogenic gene expression of BMSCs cultured on different scaffolds were investigated. The results showed that 3DP PLA/HA scaffolds stimulated the proliferation, ALP activity and osteogenesis-related gene expression of BMSCs.

Seeding efficacy with a cell suspension depends on surface roughness, volume and pore size. The 3DP PLA/HA scaffolds exhibited a lower capacity to accommodate cell suspensions during seeding when compared to β-TCP and DBM. This effect can be attributed to the relative smooth surface and larger pore size. However, the cell attachment rate could increase over 60% and the later studies, which reminded its suitability for bone regeneration in vivo, have shown the cell adhesion efficacy of 3DP PLA/HA scaffolds.

To provide a better environment for cell proliferation, HA was used as the filler material for preparing polymeric-biomaterial scaffolds. The results reveal that the 3DP PLA/HA substitutes lead to better cell proliferation than β-TCP, and DBM as an additive bioactive HA can improve the osteoconductivity of the scaffold [52].

However, the ALP levels of cells cultured on three sorts of scaffold have a different trend. The ALP production in 3DP PLA/HA is higher than in β-TCP and partially DBM on day 7. While the ALP level of partially DBM increased between days 7 and 14, and over a period of 14 days, the group in partially DBM has the highest ALP activity. For the 3DP PLA/HA scaffold, the acidity catabolite of PLA can be neutralized by the alkalinity catabolite of HA, which could maintain a relatively stable situation for a gradual increase in ALP levels [53]. β-TCP is a biodegradable inorganic bone substitute that can release various concentrations of calcium and phosphate ions. Many studies demonstrated that this release of free calcium and inorganic phosphate ions significantly reduces the ALP activities of BMSCs [54].

As for partially DBM, the ALP activity following seven days of cocultivation with relatively inaccessible BMSCs exposes osteogenic factors in the demineralization process [55]. However, the ability of DBM scaffolds could support an increased ALP activity following 14 days because osteogenic factors are released from the scaffolds in a time-dependent manner due to degradation by matrix [26]. This interpretation could therefore potentially explain the ability of different scaffolds to support different levels of ALP activity.

Additionally, the OPN and COL-1 levels increased significantly more in the DBM materials than in3DP PLA/HA and β-TCP during 14 days of cultivation, due to its content of osteogenic factors, including BMPs and other osteogenic non-collagenous proteins [26]

To explore the potential clinical applications of 3DP PLA/HA scaffolds, critical-sized calvarial defects were created to compare bone formation after the implantation of three grafts. Although some cases of foreign body reaction and inflammation were reported upon PLA implantation, the polymer is considered to have an overall satisfactory biocompatibility [56]. In our study, there was no significant difference in local cellular inflammatory reaction between the biomaterials implantation group and the non-treated group in the hematology analysis. The 3DP PLA/HA scaffolds and the other scaffolds have favorable biocompatibility *in vitro* and *in vivo*.

Degradation is a critical property of these biomaterials. An ideal biodegradable material should be comparable to the rapid replacement rate by the new bone formation, in order for the material to provide sufficient support while leaving space for tissue growth [57, 58]. Among the three types of scaffold, the degradation rate of the 3DP PLA/HA scaffold is relatively faster because this scaffold was rich in low molecular weight PLA. Lower molecular weight materials degrade faster [59]. Although the 3DP PLA/HA scaffold with the fastest degradation does not improve new bone formation, the amount of bone formation is higher than with the DBM scaffold. The β-TCP substitutes promoting newly bone formation in the highest level can not be restorable rapidly in all three materials that would hinder the replacement of new bone more or less.

Each of these materials has distinctive characteristics and advantages, and it remains a challenge to design a scaffold that can mimic the three-dimensional characteristics of autograft bone tissue.

## 5. Conclusions

In our study, 3D printed PLA/HA scaffolds have been successfully fabricated. Compared to β-TCP and partially DBM, 3D printed PLA/HA scaffolds exhibited good biocompatibility and bioactivity *in vitro*. When evaluated by a critical-size rat calvarial defect model *in vivo*, 3D printed PLA/HA scaffolds have the features of little inflammation response, relatively larger resorption rate and superior osteoinductive activity to enhance bone formation in this three different bone substitute materials. Therefore, these 3D printed PLA/HA scaffolds might be a promising candidate for bone defect repair.

## Funding

This study was supported by the National Natural Science Foundation of China [No. 81272132] and the Institutional Animal Experiment Department Shanghai Jiao Tong University School of Medicine.

## Disclosure statement

No potential conflict of interest was reported by the authors.
